# Bladder Cancer Invading the Prostate and Penis and Multiple Bone Metastases Showing Significant Improvement after a Short-Term Pembrolizumab Therapy following Radiation and Gemcitabine and Cisplatin Therapy Leading to a Pathologically Complete Remission

**DOI:** 10.1155/2024/7525757

**Published:** 2024-05-27

**Authors:** Kyohei Ishida, Akira Ogose, Gen Kawaguchi, Go Hasegawa, Yohei Ikeda, Noboru Hara, Tsutomu Nishiyama

**Affiliations:** ^1^ Department of Urology Uonuma Institute of Community Medicine Niigata University Medical and Dental Hospital, Minamiuonuma, Niigata, Japan; ^2^ Department of Orthopedic Surgery Uonuma Institute of Community Medicine Niigata University Medical and Dental Hospital, Minamiuonuma, Niigata, Japan; ^3^ Department of Radiotherapy Uonuma Institute of Community Medicine Niigata University Medical and Dental Hospital, Minamiuonuma, Niigata, Japan; ^4^ Department of Pathology Uonuma Institute of Community Medicine Niigata University Medical and Dental Hospital, Minamiuonuma, Niigata, Japan; ^5^ Department of Diagnostic Radiology Uonuma Institute of Community Medicine Niigata University Medical and Dental Hospital, Minamiuonuma, Niigata, Japan

## Abstract

A 65-year-old man was diagnosed with bladder cancer invading the prostate and penis and multiple bone metastases. He underwent palliative radiation (30 Gy/10 fr) through vertebral bones (Th3 and Th12-L5) and pelvic bones for pain control. The patient received pembrolizumab therapy after three courses of gemcitabine and cisplatin therapy. CT four weeks after starting pembrolizumab therapy showed that both the primary and metastatic lesions had notably reduced in size, and no new lesion was detected. He subsequently fell, resulting in a femoral neck pathological fracture, and underwent hemiarthroplasty. Pathological examination of the pathological fracture site revealed no residual tumor tissue.

## 1. Introduction

Metastatic urothelial cancer is known to be difficult to treat, and even after the introduction of cisplatin-based chemotherapy and immune checkpoint inhibitors (ICIs), the median patient survival remains less than 15 months [1, 2]. Bone metastasis is relatively common in patients with urothelial cancer. The median survival time was reported to be 6.2 months after diagnosis of bone metastasis [3].

We treated a patient with bladder cancer that had spread to the prostate, penis, and multiple bones. The patient showed significant improvement after a short-term pembrolizumab therapy following radiation and gemcitabine and cisplatin therapy, ultimately leading to a complete remission based on pathological findings.

## 2. Case Presentation

**Figure 1:**
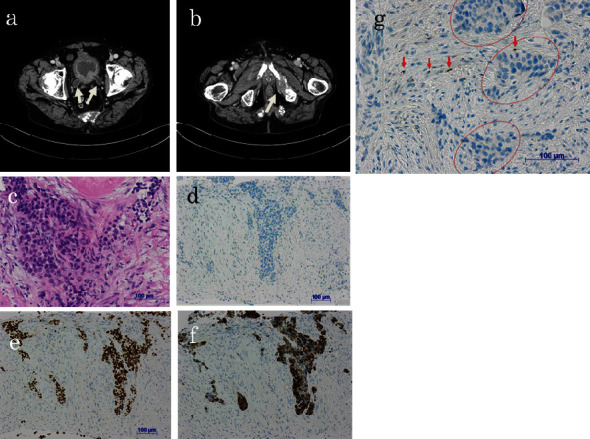
CT findings before treatments showed an invasive bladder tumor with (a) bilateral hydroureters (arrows) and (b) osteolytic metastasis (arrow). Pathological findings of needle biopsy of bladder and prostate lesions revealed luminal-type urothelial carcinoma ((c) hematoxylin and eosin), (d) androgen receptor-negative, (e) GATA3-positive, (f) CK20-positive, and (g) PD-L1 negative. There were almost no tumor-infiltrating lymphocytes within the tumor tissue ((g) circles). Internal control histiocytes are PD-L1 positive ((g) arrow).

A 65-year-old man had been a heavy smoker and had smoked at least 20 cigarettes every day since the age of 20. He suffered from back and pelvic pain and penile swelling with pain for five months. He suffered from gross hematuria for two months. He visited our hospital in December 2022. He suffered from priapism and could not undergo cystoscopy. Computed tomography (CT) and magnetic resonance imaging (MRI) revealed a bladder tumor invading the prostate and penile base, and multiple bone metastases with pelvic and vertebral bones (Th3 and Th12-L5) (Figure 1). He received a needle biopsy of the bladder and prostate lesions transperineally. The pathological findings revealed urothelial carcinoma (Figure 1). He underwent palliative radiation (30 Gy/10 fr) through vertebral (Th3 and Th12-L5) and pelvic bones for pain control.

**Figure 2:**
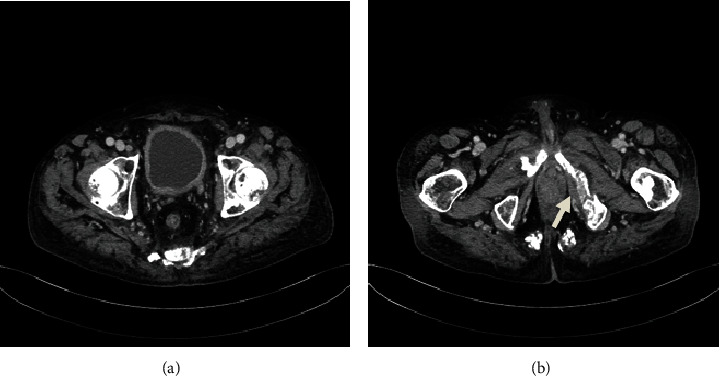
CT findings four weeks after starting pembrolizumab therapy revealed a significant reduction in the size of the primary lesion (a), as well as a decrease in osteolytic lesions at the bone metastatic sites with a shift towards osteoblastic change (b, arrow).

The patient initially received gemcitabine and cisplatin (GC) therapy after palliative radiation. After completing two courses of GC therapy, there was little change in the size of both primary and metastatic lesions compared to before chemotherapy. He also suffered from myelosuppression associated with GC therapy. We therefore discontinued GC therapy after only three courses. He began to receive pembrolizumab therapy (200 mg once every 3 weeks). Due to his increasing back pain, a CT scan was conducted four weeks after starting pembrolizumab therapy to assess the early treatment response. CT showed that both the primary and metastatic lesions had notably reduced in size, and no new lesion was detected (Figure 2).

**Figure 3:**
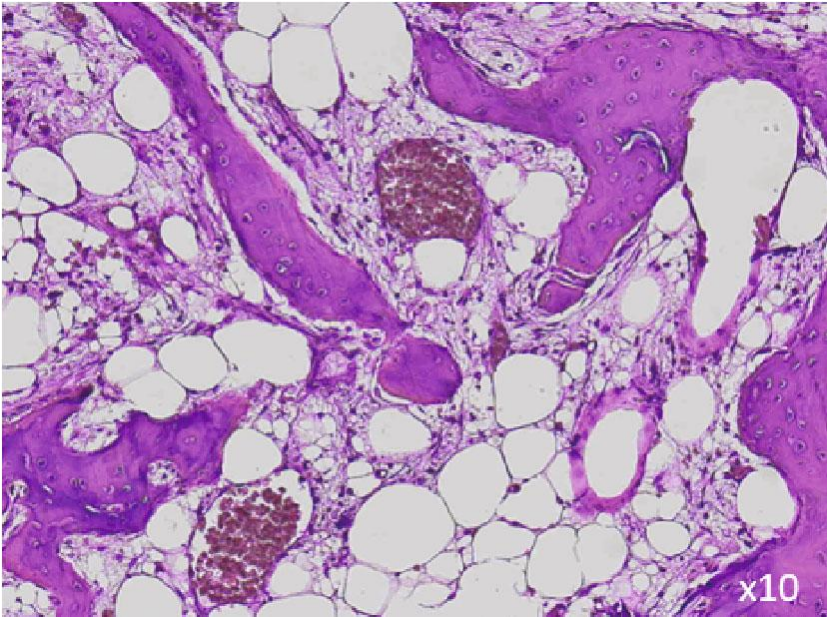
Pathological findings of fracture lesion revealed no residual cancer tissue with osteoblastic change.

In September 2023, he fell, resulting in a pathological femoral neck fracture, and received hemiarthroplasty. Pathological examination of the fracture site revealed no residual tumor tissue (Figure 3). He is currently undergoing pembrolizumab therapy (400 mg once every 6 weeks since September 2023), and as of March 2024, no recurrence has been observed.

## 3. Discussion

We treated a patient with a history of heavy smoking who had bladder urothelial cancer, prostate and penile invasion, and multiple bone metastases. He showed significant improvement after receiving a short-term pembrolizumab therapy following radiation and GC therapy. The patient achieved complete remission, which was confirmed pathologically.

Bone is reportedly the most common site for bladder cancer metastases [3]. Molecular subtypes of urothelial bladder cancer are significantly correlated with specific metastatic sites. Sjödahl et al. observed a significant depletion of bone metastases in the basal/squamous molecular subtype, whereas the urothelial-like subtype showed more metastases to bone [4]. In our case, pathological findings showed luminal-type urothelial carcinoma with GATA3, CK20, and uroplakin-positive.

Treatment of patients with advanced urothelial cancer is rapidly evolving, with advancements such as immunotherapy using ICIs and enfortumab vedotin, an antibody-drug conjugate [2, 5]. However, cisplatin-based chemotherapy remains the current standard treatment for metastatic urothelial carcinoma [1]. Cisplatin-based chemotherapy is recommended for eligible patients based on overall survival and progression-free survival benefits, as well as the objective response rates observed in randomized trials and clinical practice guidelines [6]. However, in the present patient after completing two courses of GC therapy, both the primary and metastatic lesions showed minimal change in size following the chemotherapy. He also suffered from myelosuppression associated with GC therapy, so we decided to discontinue GC therapy after only three courses.

The use of ICIs such as pembrolizumab has improved the survival outcomes of patients with metastatic urothelial cancer [2]. Pembrolizumab led to significantly longer overall survival (approximately 3 months) than chemotherapy in patients with previously treated metastatic urothelial cancer; however, achieving complete remission is rare. Several reports indicate that anti-PD-1 or anti-PD-L1 antibody therapy may enhance radiotherapy-induced antitumor immunity [7]. Recent reports indicate that a higher number of tumor-infiltrating lymphocytes are believed to predict a positive response to ICIs [8]. In the present patient, the cancer tissue did not express PD-L1, and there were almost no tumor-infiltrating lymphocytes within the tumor tissue (Figure 1). Despite this, both the primary and metastatic lesions had noticeably reduced in size after receiving two courses of pembrolizumab therapy (200 mg once every 3 weeks) (Figure 2). Subsequently, the metastatic bone site achieved pathologically confirmed complete remission (Figure 3).

In the case of lung cancer, a positive correlation has been reported between a history of smoking and the effectiveness of ICI treatment [9]. The present patient had a heavy smoking, consuming at least 20 cigarettes daily for over 40 years. Patients with a history of smoking may experience benefits from ICI treatment compared to those who have never smoked, in cases of bladder cancer.

Radiotherapy plus ICIs can potentially induce synergistic antitumor immune responses [10–12]. The combination of radiation therapy and ICI may enhance survival outcomes after pembrolizumab treatment in patients with advanced urothelial cancer. Nevertheless, the ideal timing, dosages, and sequence of radiation therapy remain poorly understood. A case involving the combined treatment of palliative radiotherapy and immunotherapy is reported to have resulted in an effective response, benefiting a patient with urothelial cancer [13]. The present patient received palliative radiation for pain control in the vertebral and pelvic bones, without involving the primary tumor. Regarding chemotherapy, there are several reports indicating its effectiveness when combined with immunotherapy [14]. The present patient underwent three courses of GC therapy. The present patient's medical conditions appear to have significantly improved after a brief course of pembrolizumab therapy, possibly due to a combination of factors such as smoking history, radiation therapy, and chemotherapy.

## 4. Conclusion

The present patient's disease conditions, including invasive bladder cancer and multiple bone metastases, have shown significant improvement after a short-term pembrolizumab therapy, leading to a pathologically complete remission. This improvement may be attributed to a combination of factors, such as smoking history, radiation therapy, and GC therapy.
